# ^1^H-NMR-Based Metabolomic Analysis of Cerebrospinal Fluid From Adult Bilateral Moyamoya Disease: Comparison With Unilateral Moyamoya Disease and Atherosclerotic Stenosis

**DOI:** 10.1097/MD.0000000000000629

**Published:** 2015-05-01

**Authors:** Jin Pyeong Jeon, Taeho Yun, Xing Jin, Won-Sang Cho, Young-Je Son, Jae Seung Bang, Hyun-Seung Kang, Chang Wan Oh, Jeong Eun Kim, Sunghyouk Park

**Affiliations:** From the Department of Neurosurgery (JPJ), Hallym University College of Medicine, Chuncheon; College of Pharmacy (TY, XJ, SP), Seoul National University; and Department of Neurosurgery (JEK, W-SC, Y-JS, JSB, H-SK, CWO), Seoul National University College of Medicine, Seoul, Korea.

## Abstract

Although metabolomics has been increasingly used to observe metabolic pattern and disease-specific metabolic markers, metabolite profiling for moyamoya disease (MMD) has not yet been done in adults. This study investigated cerebrospinal fluid (CSF) metabolites specific to bilateral MMD (B-MMD) and compared them to those of unilateral MMD (U-MMD) or atherosclerotic stenosis with hydrogen-1 nuclear magnetic resonance spectroscopy to identify metabolic biomarkers associated with MMD in adults.

CSF samples of B-MMD (n = 29), U-MMD (n = 11), and atherosclerotic cerebrovascular disease (ACVD) (n = 8) were recruited. Principal component analysis, partial least square discriminant analysis, and orthogonal projections to latent structure discriminant analysis (OPLS-DA) were done for the comparisons. Diagnostic performance was acquired by prediction of 1 left-out sample from the distinction model constructed with the rest of the samples.

B-MMD showed an increase in glutamine (*P* < 0.001) and taurine (*P* = 0.004), and a decrease in glucose (*P* < 0.001), citrate (*P* = 0.002), and myo-inositol (*P* = 0.006) than those in ACVD. U-MMD showed a higher level of glutamine (*P* = 0.005) and taurine (*P* = 0.034), and a lower level of glutamate (*P* < 0.004) than those in ACVD. No difference at the metabolite level was observed between B-MMD and U-MMD. Cross-validation with the OPLS-DA model showed a high accuracy for the prediction of MMD.

The results of the study suggest that a metabolomics approach may be helpful in confirming MMD and providing a better understanding of MMD pathogenesis. Elevated glutamine in the CSF may be associated with MMD pathogenesis, which was different from ACVD.

## Abstract 2

Supplemental Digital Content is available in the text

## INTRODUCTION

Typical moyamoya disease (MMD) is characterized by a bilateral progressive steno-occlusive lesion of the terminal internal carotid artery or proximal anterior cerebral artery (ACA) and/or middle cerebral artery (MCA).^1^ Although studies concerning geographical distribution, sex preference, clinical manifestation, age-specific characteristics, and basic researches including genomic and proteomics approaches have been done extensively, the etiologies of the disease still remain unclear.^2^ In addition, the prevalence of unilateral MMD (U-MMD) and atherosclerotic change with age can increase the difficulty of understanding the pathogenesis of MMD in an adult population.

Diagnosis of MMD is usually confirmed with angiographic findings. Accordingly, MMD should be considered in the differential diagnosis of moyamoya-like vascular change. In particular, atherosclerotic steno-occlusive arterial lesions with secondary collateral changes should be differentiated from MMD in adults. The reasons for this are that clinical presentation, treatment policy, and disease progression of adult MMD are different from those of atherosclerotic cerebrovascular disease (ACVD). For adult MMD, the prevalence rate of hemorrhage has been reported to range from 10% to 40%,^1,3^ and the combined revascularization surgery consisting of direct anastomosis between the superficial temporal artery-MCA with encephalodurogaleosynangiosis has been an effective treatment method.^4^ Disease progression rates of 17.4% per hemisphere and 23.8% per patient have been reported.^5^ In contrast, combined revascularization surgery is not a routine treatment modality for patients with ACVD who usually present with cerebral ischemia. An angiographic progression rate of 32.5% has been reported in patients with symptomatic MCA stenosis.^6^

Metabolomics has recently emerged as a promising new area to observe metabolic pattern and disease-specific metabolic markers. Studies on disease-specific metabolites for atherosclerosis,^7^ coronary artery disease (CAD),^8^ intracranial tumors^9,10^ and cerebral infarctions^11^ have been reported. However, to the best of our knowledge, metabolite profiling for adult bilateral MMD (B-MMD) has not yet been done. Because cerebrospinal fluid (CSF) has close contact with the extracellular fluid (ECF) of the brain, we hypothesized that metabolite analysis at the CSF level may provide a better understanding of MMD pathogenesis. In this study, we investigated the CSF metabolites of B-MMD and compared them to those of U-MMD and atherosclerotic stenosis with hydrogen-1 nuclear magnetic resonance (^1^H-NMR) spectroscopy to identify metabolic biomarkers associated with MMD, which could be helpful in confirming MMD and providing a better understanding of MMD pathogenesis in adults.

## METHODS

This study and the sampling of CSF were approved by the institutional review board of Seoul National University Hospital (H-1406-117-591). This prospective analysis was conducted in patients who underwent surgical revascularization for ischemic symptoms in B-MMD (n = 29), U-MMD (n = 11), and ACVD (n = 8) of the intracranial arteries from April 2011 to March 2012 at a single institution. Their medical information including sex, age, hypertension (HTN), diabetes mellitus (DM), hyperlipidemia, smoking, pregnancy, and radiologic data were reviewed. Patients who took medication for underlying diseases including HTN, DM, and hyperlipidemia or patients with uncontrolled underlying diseases were excluded from the analysis. Written informed consent was obtained from all patients or their families before entering the study.

### CSF Sample Preparation and ^1^H-NMR Spectroscopy

**FIGURE 1:**
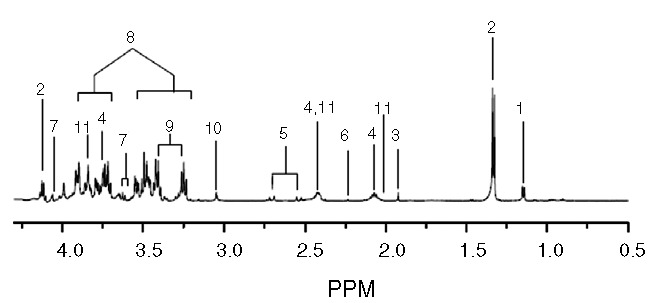
Representative 600-MHz ^1^H-NMR spectra of CSF. Key for CSF is as follows: 1, Isopropanol; 2, lactate; 3, acetate; 4, glutamine; 5, citrate; 6, 2-aminoadipate; 7, myo-inositol; 8, glucose; 9, taurine; 10, creatine; 11, glutamate. CSF = cerebrospinal fluid, ^1^H-NMR = hydrogen-1 nuclear magnetic resonance, PPM = ??.

CSF samples ranging from 5 to 15 mL were collected from the cortical subarachnoid space and stored at −80°.^12^ For the nuclear magnetic resonance (NMR) analysis, CSF samples were thawed at room temperature, and 450 μL of CSF was mixed with 50 μL of D_2_O containing 0.25% sodium-3-trimethylsily-[2,2,3,3-^2^H_4_]-1-propionate (TSP) as an internal standard and placed in a 5-mm NMR tube. All 1-dimensional spectra of the CSF samples were measured with an NMR spectrometer (Avance 600; Bruker BioSpin, Rheinstetten, Germany) operating at a proton NMR frequency of 600.13 MHz. The NMR experiment was performed at the Seoul National University's National Center for Inter-University Research Facilities. The spectra were apodized with an exponential function (1 Hz), Fourier transformed, phased, and baseline-corrected manually. The signals were referenced to the TSP signal at 0.00 ppm and normalized against the total integration values. To avoid artifacts in the downstream analyses, ethanol and water peaks were eliminated. The spectra were binned at every 0.03 ppm intervals to reduce the complexity of the NMR data for pattern recognition. The normalization and binning were accomplished with an in-house-built Perl program. The parameters of the acquisition were fundamentally the same as those previously reported.^13,14^ The metabolites were confirmed with Chenomx (spectral database; Chenomx, Edmonton, Alberta, Canada) by fitting the experimental spectra to those in the database and comparing them with spectra from standard compounds. The spectra of the CSF were feathered with conspicuous metabolites, shown by the metabolite key in Figure 1. Because proteins with large molecular weights do not contribute much to NMR signals due to the rapid relaxation of their signals, protein signals were not considered in the analyses.

### Statistical Analysis and Validation

Statistical analysis was done with SIMCA-P 11.0 (Umetrics, Umea, Sweden) and R (from R Project for Statistical Computing). Chenomx (Spectral database) and an in-house-built database were used to identify metabolites. After the principal component analysis (PCA) was carried out, partial least square discriminant analysis (PLS-DA) and Orthogonal projections to latent structure discriminant analysis (OPLS-DA) were done to identify latent patterns and to compare the whole metabolite profile. Class discrimination models were built until the cross-validated predictability value did not increase significantly to prevent overfitting the statistical model. Diagnostic performance was estimated by the prediction of randomly selected left-out samples (one third of the total samples) from the distinction model built using the rest of the samples.^15–17^ As a result, 10 of 29 from the B-MMD samples, 5 of 11 from the U-MMD samples, and 3 of 8 from the ACVD samples were randomly selected as prediction samples to evaluate the diagnostic performance of the model. Moreover, a prior cut off value of 0.5 was applied to evaluate the prediction results. The cut off value was chosen, as each group was assigned to either 0 or 1, and the group membership of the sample was predicted by the multivariate model. The middle value (0.5) was chosen; however, any value that gives the best combination of sensitivity and specificity can be selected in practice.^17,18^

### Concentration Measurement of the Nitric Oxide Metabolome (Nitrite)

To quantitate the amount of nitric oxide (NO) within the CSF, the concentrations of nitrite (NO_2_^−^), which is a stable metabolome of NO, and nitrate (NO_3_^−^), which is oxidized and originates from NO, were measured with a NOx analyzer (ENO-20; Eicom, Kyoto, Japan). ENO-20 is a device that analyzes and measures the ion value of nitrite and nitrate within a liquid sample through the combined application of the diazo coupling method and high-performance liquid chromatography and separates the 2 with high sensitivity. When a sample is added to the ENO-20, it is filtered by the guard column, and each of the mixed components is trapped on the separation column. During the trapping of mixed components within the sample, NO_2_^−^ and NO_3_^−^ are separated, and the concentration is calculated. NO_2_^−^ and NO_3_^−^ as individual samples from the separation column are moved to a reduction column. First, NO_2_^−^ enters the reduction column followed by NO_3_^−^. NO_3_^−^ is reduced to NO_2_^−^ as a result of the reaction with cadmium-copper within the reduction column and is reduced copper. The reduced NO_2_^−^ from the NO_3_^−^ is mixed with the reaction solution to produce the diazo compound, and the overall concentration of the diazo compound is measured at a wavelength of 540 nm. To measure the concentration of the NO metabolome, the sample was pretreated with methanol at twice the volume of the CSF, and only the supernatant was taken for the analysis of NO metabolome after 30 seconds of vortexing and 20 minutes of centrifugation at 13,200 rpm and 4°C. An Autosampler 3023 (NANOSPACE SI-2; Shiseido, Tokyo, Japan) was used for sample injection into the NOx analyzer. Ten microliters of sample per measurement were injected and separated into NO_2_^−^ and NO_3_^−^ after going through each column for 10 minutes. The measurement was done twice for each sample. The analysis of the values measured with the NOx analyzer was done with the S-MC21 (data processing system, Shiseido) program.

## RESULTS

### Enrolled Patients

A total of 48 patients were included in the analysis. The number of patients for each disease was as follows: B-MMD, n = 29 (60.4%); U-MMD, n = 11 (22.9%); ACVD, n = 8 (16.7%). Detailed information on the demographical characteristics is shown in Table 1. The mean ages of the MMD patients were 32.4 ± 8.2 years for B-MMD, 39.9 ± 9.5 years for U-MMD, and 53.1 ± 8.1 years for ACVD. A male dominance was observed in ACVD (n = 4, 50.0%) compared with B-MMD (n = 6, 20.7%) and U-MMD (n = 2, 18.2%). The surgical timing did not differ significantly among B-MMD (14.8 months [2.0–45.0]), U-MMD (11.9 months [2.0–46.0]), and ACVD (8.2 months [2.0–24.0]) (*P* = 0.14).

**TABLE 1:**
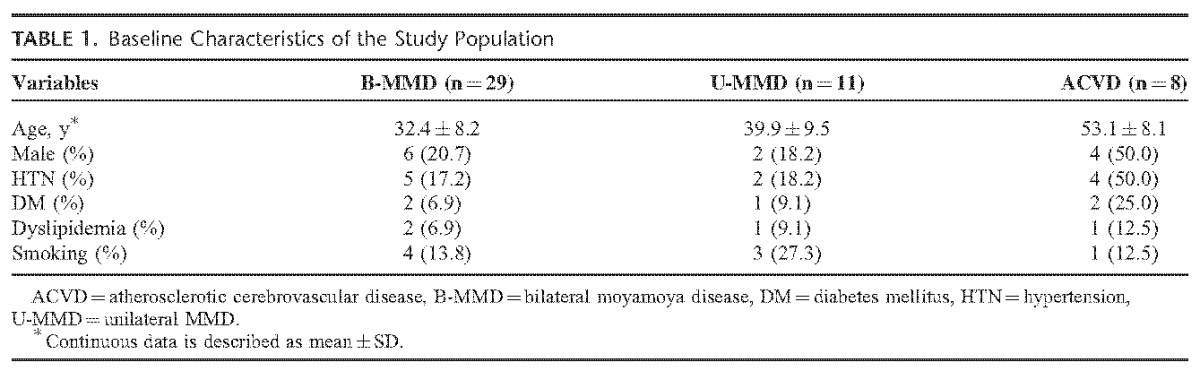
Baseline Characteristics of the Study Population

### Multivariate Analysis with PCA, PLS-DA, and OPLS-DA

**FIGURE 2:**
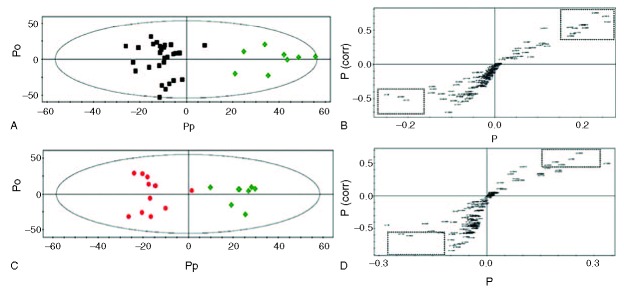
OPLS-DA and S-plot analysis between B-MMD and ACVD (A and B), and U-MMD and ACVD (C and D). Potential markers that were significantly different between the 2 groups are enclosed in dotted boxes. ▪, B-MMD; ●, U-MMD; ♦, ACVD. ACVD = atherosclerotic cerebrovascular disease, B-MMD = bilateral moyamoya disease, OPLS-DA = Orthogonal projections to latent structure discriminant analysis, U-MMD = unilateral MMD.

First, PCA was done to investigate the intrinsic variation in the CSF dataset. However, the score plots exhibited nonseparation between B-MMD and ACVD (supplemental Figure 1A, http://links.lww.com/MD/A232), and U-MMD and ACVD (supplemental Figure 1C, http://links.lww.com/MD/A232). To investigate the statistical meaning of those signals and to exclude possible intragroup confounding variables not related to the group difference, PLS-DA and OPLS-DA were applied, and the overall metabolites were compared in each group (supplemental Table 1, http://links.lww.com/MD/A232). The result of the PLS-DA is shown in the supplementary Figures 1C and D (http://links.lww.com/MD/A232). The OPLS-DA models (R2Y = 0.866, Q2 = 0.602) and score plots represent an obvious differentiation between the 2 groups: B-MMD and ACVD (Figure 2A and B). The CSF of the B-MMD seems to have higher levels of isopropanol, acetate, glutamine, 2-aminoadipate, taurine, and creatine, and a lower level of lactate, citrate, myo-inositol, glucose, and glutamate compared with ACVD. Regarding U-MMD and ACVD, the score plots from the OPLS-DA models (R2Y = 0.898, Q2 = 0.241) show a significant differentiation between the 2 groups of U-MMD and ACVD (Figure 2C and D). The CSF of the U-MMD seemed to have increased levels of acetate, glutamine, glucose, myo-inositol, taurine, and creatine, and decreased levels of isopropanol, lactate, citrate, 2-aminoadipate, and glutamate compared with ACVD. Subsequently, a combination of statistical approaches was used to identify the significance of these metabolites.^11^ Accordingly, we assigned biomarkers using S-plot,^19–21^ Chenomx, *t* test, and the associated *P* values. The measured glutamine (*P* < 0.001) and taurine (*P* = 0.004) in B-MMD were significantly higher than those in atherosclerosis. Atherosclerotic stenosis showed a meaningful higher level of glucose (*P* < 0.001), citrate (*P* = 0.002), and myo-inositol (*P* = 0.006). Compared with the levels of the metabolites between U-MMD and ACVD, the measured glutamine (*P* = 0.005) and taurine (*P* = 0.034) were significantly higher in U-MMD (Table 2). Regarding B-MMD and U-MMD, no discriminate difference in the metabolite level was observed (data not shown).

**TABLE 2:**
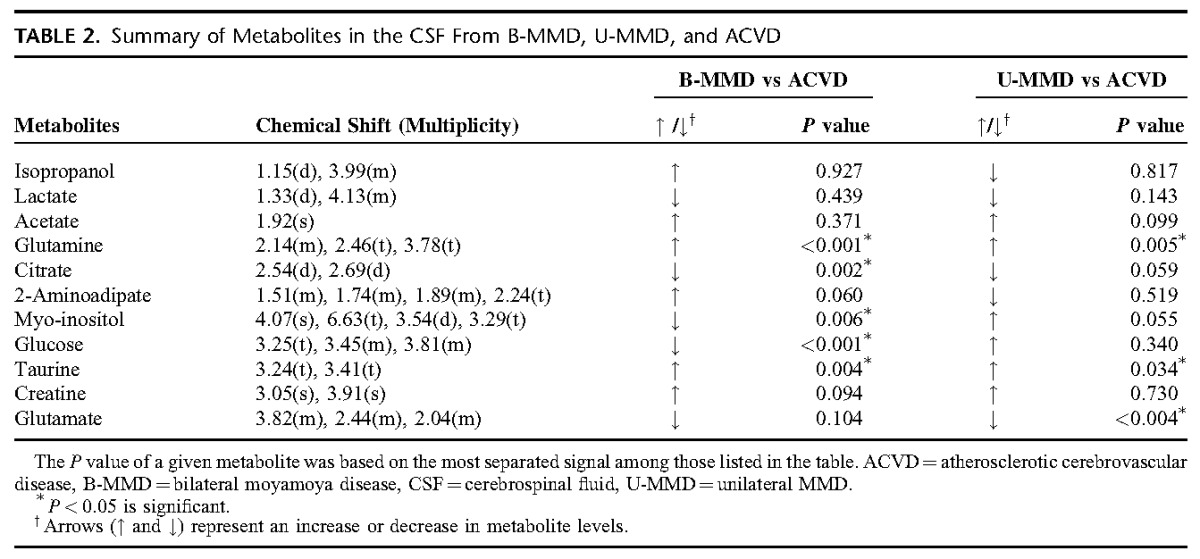
Summary of Metabolites in the CSF From B-MMD, U-MMD, and ACVD

### Diagnostic Performance with Cross Validation

**FIGURE 3:**
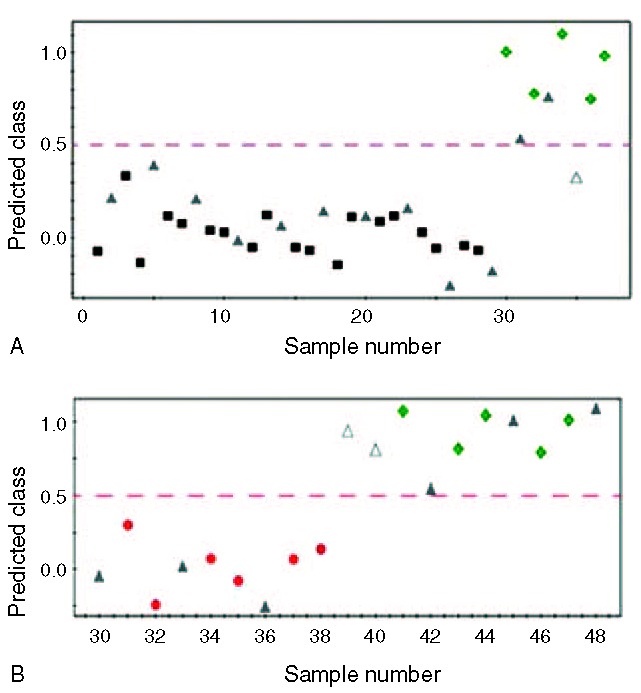
Y-predicted scatter plot of an OPLS-DA model between B-MMD and ACVD (A), and U-MMD and ACVD (B). The OPLS-DA model of B-MMD and ACVD patients had a slightly higher goodness of fit (R2X = 0.424, Q2 = 0.531) than that of U-MMD and ACVD (R2X = 0.647, Q2 = 0.252). ▪, B-MMD; ●, U-MMD; ♦, ACVD; ▴; test set; ▵; mispredicted sample; Y predicted cutoff of 0.5. ACVD = atherosclerotic cerebrovascular disease, B-MMD = bilateral moyamoya disease, MMD = moyamoya disease, OPLS-DA = Orthogonal projections to latent structure discriminant analysis, U-MMD = unilateral MMD.

To make sure that the performance of the OPLS-DA model is reliable, cross-validation was conducted. One third of the CSF samples were randomly chosen from each group, and the prediction model was formed with the rest of the samples. The OPLS-DA model for B-MMD and ACVD had a slightly lower goodness of fit (R2X = 0.424, Q2 = 0.531) compared with the model for U-MMD and ACVD (R2X = 0.647, Q2 = 0.252). For the differential diagnosis between B-MMD and ACVD, 10 of the 10 blind B-MMD samples were correctly predicted as B-MMD, whereas 2 of the 3 blind ACVD samples were correctly predicted as ACVD (Figure 3A). For U-MMD versus ACVD, 3 of the 5 blind U-MMD samples were correctly predicted as U-MMD, whereas 3 of the 3 blind ACVD samples were correctly predicted to be ACVD (Figure 3B). Because we did not compare the difference between patients with diseases and normal controls, the sensitivity and specificity of the diagnosis is not relevant. Still, overall differential diagnostic performance can be estimated to be 12 of the 13 (92%) for B-MMD versus ACVD and 6 of the 8 (75%) for U-MMD and ACVD.

## DISCUSSION

This investigation suggests that higher CSF levels of glutamine and taurine may be associated with the pathogenesis of MMD in both bilateral and unilateral cases of adult MMD. Regarding B-MMD and U-MMD, no significant difference at the metabolite levels was observed.

Higher expression of vascular endothelial growth factor (VEGF)/VEGF receptor expression of endothelium,^22^ nestin positive, which is a marker for newly formed endothelial cell, in the affected vessels,^22,23^ increased the number of circulating endothelial progenitor cells,^24^ and smooth muscle cell (SMC) proliferation and migration and inflammatory processes are known to be associated with MMD development. Regarding atherosclerosis, endothelial dysfunction, inflammatory change, vascular SMC proliferation, and arterial wall remodeling were related to the pathogenesis of atherosclerosis.^25^ The causes of endothelial dysfunction include hypercholesterolemia, oxidative stress, hyperglycemia, and inflammatory change in patients with atherosclerosis.^25,26^

Glutamine is an amino acid that has various biochemical functions such as ammonium synthesis, an energy source during fasting, and a donor of nitrogen and carbon.^27–29^ The association between glutamine and MMD has not been well studied. Wurtz et al^8^ reported that glutamine was associated with high intima-media thickness of the carotid arteries and CAD. Shah et al^30^ showed that the glutamate/glutamine level and tyrosine were significant metabolites for diagnosing CAD. Glutamine supplementation alleviates vasculopathy in a mouse model of endothelial cell dysfunction triggered by the administration of L-N-ω-methylarginine.^31^ In their study, a defect in acetylcholine inducing the relaxation of the aortic rings was prevented by glutamine supplementation. In our study, glutamine in the CSF from B-MMD or U-MMD was significantly higher than that of ACVD. When considering a more extensive involvement of arterial stenosis in MMD than that in ACVD, a higher glutamine level may be related to MMD pathogenesis through a more extensive endothelial dysfunction and thickened intima by abnormal SMC proliferation. In addition, cerebral infarct was related with a decreased level of plasma glutamine.^11^ Accordingly, significantly increased glutamine in MMD could be a distinguishing metabolite from ACVD or cerebral infarct. However, the precise mechanism of glutamine on the development of MMD remains unclear. Arnal et al^32^ suggested that l-glutamine may attenuate receptor-mediated NO release. Because NO plays a role in the antiatherogenic effect such as inhibition of SMC proliferation^33^ and platelet aggregation and adhesion,^34^ as well as maintaining vascular tone and decreasing hemodynamic stress,^35^ an increase of glutamine could be related to endothelial dysfunction though a reduction of NO release.^32^ To investigate the relationship between MMD and the NO level, we indirectly estimated them by measuring the sum of the NO_2_ and NO_3_ using the NOx analyzer (Supplemental Figure 2, http://links.lww.com/MD/A232). The measured NO level was 1.99 ± 0.63 μM (mean ± SD) in B-MMD, 1.39 ± 0.45 μM in U-MMD, and 1.94 ± 0.55 μM in ACVD. U-MMD showed significantly lower NO levels than that of ACVD. However, the NO level in B-MMD did not differ significantly from that in ACVD. Therefore, an increase of glutamine could not affect typical B-MMD pathogenesis through NO reduction in adults. Noda et al^36^ also reported that NO concentrations in the CSF were elevated in MMD, which could reflect abnormal collateral vessel formation. Accordingly, an elevated NO level in MMD could be a compensatory mechanism related to progressive stenosis of the distal internal carotid artery and proximal MCA or ACA. Therefore, in the future, studies on the role of glutamine in the MMD pathogenesis are necessary.

Taurine is an amino acid, which has various biochemical functions such as bile acid conjugation, membrane stabilization, antioxidant, osmoregulation, and iron regulation.^26,37^ Terauchi et al^38^ reported the presence of taurine in the vascular endothelium and SMCs. The taurine level in the intima-media of the arterial wall is higher than that of the plasma level.^39^ Taurine supplementation has shown beneficial effects in preventing atherosclerosis.^40^ The mechanisms of taurine in decreasing atherosclerosis include antioxidant, anti-inflammation, inhibition of SMC proliferation, and increasing hepatic cholesterol metabolism.^26,40,41^ Balkan et al^42^ reported that taurine attenuated oxidative stress and cholesterol accumulation in rabbits with a high-cholesterol diet. The decrease of vascular SMC proliferation and neointimal hyperplasia were observed after taurine supplementation.^40^ The results of our study showed that increased taurine in B-MMD may be a discriminator metabolite that was different from ACVD. However, the difference in dietary behavior and age at diagnosis (B-MMD, 32.4 ± 8.2 years vs ACVD, 53.1 ± 8.1 years) can be a concern when interpreting the results. That is because the serum taurine level is mainly maintained by diet supplementation due to their low endogenous synthesis. In addition, a decrease in taurine level was observed with age progression.^39^ Accordingly, the association between an increase of taurine in the CSF level and MMD pathogenesis should be investigated further.

**FIGURE 4:**
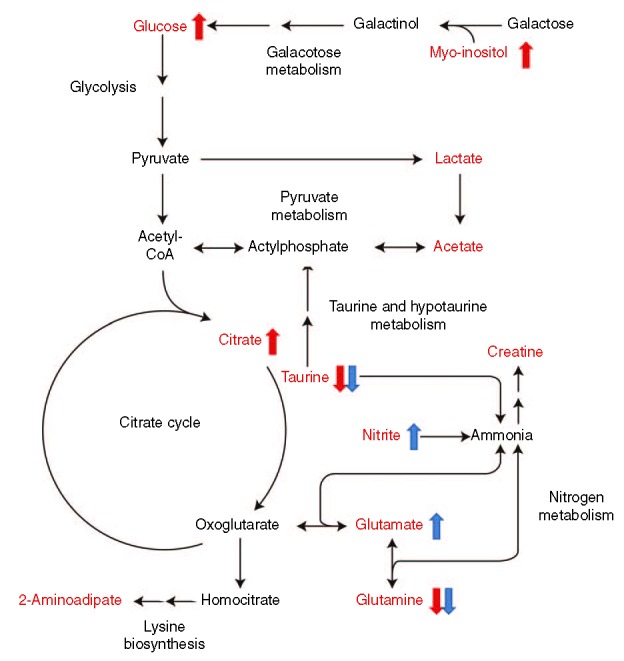
Summary of the metabolic pathway in patients with ACVD. The red characters represent the metabolites identified in this study. The metabolites with arrows are significantly changed. A red or blue arrow shows the trend of the metabolites in ACVD compared with B-MMD or U-MMD, respectively. ACVD = atherosclerotic cerebrovascular disease, B-MMD = bilateral moyamoya disease, CoA = coenzyme A, U-MMD = unilateral MMD.

B-MMD revealed a significantly lower CSF level of glucose, citrate, and myo-inositol than those of ACVD. Glucose seems to stimulate leukocyte adherence to the arterial wall and SMC accumulation.^43,44^ Renard et al^45^ reported that hyperglycemia was related to atherosclerosis development and progression. We suspected that an increased level of glucose could be attributed to an increase in the citrate, glutamate, and myo-inositol levels in patients with ACVD (Figure 4).

Glutamate is the excitatory neurotransmitter in the brain. The measured concentration of glutamate in the CSF and brain ECF was approximately <1 μmol/L and 0.5 to 2 μmol/L, respectively.^46^ Glutamate release is associated with cerebral ischemia. Davalos et al^47^ reported that an increase in glutamate in the CSF was not observed for >6 hours in patients with a stable stroke. However, patients with progressive ischemic stroke showed a sustained elevation of glutamate. Studies on therapeutic targets at the glutamate synapse have been done for patients with ischemic stroke. The protective effects of antagonists for inotropic receptors of *N*-methyl-d-aspartate^48^ or α*-*amino-3-hydroxy-5-methyl-4-isoxazolepropionic acid^49^ have been investigated. Although some animal studies has shown a neuroprotective effect,^50,51^ a definite conclusion has not been made in a cohort of stroke patients.^52^ The role of glutamate has not been well studied in MMD patients. Mendelowitsch et al^53^ reported that direct bypass between external and internal cranial arteries improved chronic ischemic deficit corresponding to a rapid decrease in the level of glutamate in the MMD patient secondary to the sickle cell disease. Accordingly, further studies are needed on glutamate in MMD patients.

Previous studies reported that U-MMD had similar characteristics of ACVD. Houkin et al^54^ showed that basic fibroblast growth factor in the CSF was low in U-MMD (mean ± SEM, 4.1 ± 1.9 pg/mL) and ACVD (6.5 ± 7 pg/mL) compared with B-MMD (54.7 ± 9.98 pg/mL). Jeon at al^12^ also reported that cellular retinoic acid-binding protein-I intensities in the CSF from U-MMD (median [25th–75th percentile], 0.91 [0.78–1.20]) were similar to that in ACVD (0.85 [0.66–1.11]) (*P* = 0.883), which were significantly lower than that of B-MMD (1.45 [0.86–2.52]). However, metabolomic analyses suggested that U-MMD and B-MMD did not differ at the metabolite level. Additionally, U-MMD also showed a higher level of glutamine (*P* = 0.005) and taurine (*P* = 0.034) than those in ACVD.

In analyzing the metabolic features that contribute to a differential diagnosis, there may be intragroup confounding factors not related to the disease groups. The OPLS-DA used in this study is a robust method that deals with these confounding variables with its inherent orthogonal signal correction features. It rotates the entire data space to maximize the metabolic difference between the groups of interest, in this case MMD versus ACVD, and the resulting predictive component shows the metabolic markers relevant to that specified difference. Other orthogonal components are related to confounding variables and do not affect the separation of the groups. Therefore, this method has been used extensively in complicated metabolomics studies,^18,55,56^ and the technical aspects have been well described.^57,58^

There are some limitations to this study. First, selection bias because of person-to-person variability could occur due to the small number of patients. In such cases, metabolomics analysis of the serum and CSF could more appropriately reflect the pathogenesis than just a simple analysis of the CSF. Verwaest et al^59^ reported metabolomics alterations in the serum and CSF of a transgenic rat model of Huntington disease. Accordingly, further studies on both serum and CSF are needed to find MMD specific biomarkers. Second, our results may be limited due to the absence of a comparison with a healthy control group. Previous studies have investigated disease-specific metabolites with a comparative analysis using a healthy control group. Comparative studies between MMD patients and healthy young adults are more accurate in finding MMD-specific metabolites but have limitations due to ethical problems. Third, the differential diagnostic performance in U-MMD versus ACVD and B-MMD versus ACVD was reasonably high. An investigation with a larger patient group will prove whether this diagnostic performance can be generally used as a reference. Fourth, differences in demographic characteristics such as age and sex can be a concern when interpreting the results. Although, MMD has shown a bimodal age distribution (peaks at ages 5–9 years and 35–39 years) and a female predominance,^60^ differentiation between the 2 diseases can be difficult in clinical situations. In a review of the literature, only a few studies have attempted to show the differences between MMD and ACVD.^12,54^ However, in those studies, the differences in the mean age (years) and the number of men were 36.4 ± 11.4 in B-MMD versus 55.0 ± 9.0 in ACVD; n = 17 (29.3%) in B-MMD versus n = 14 (66.7%) in ACVD.^12^ Accordingly, age and sex differences can be an inherent bias in studies between adult MMD and ACVD. Nevertheless, further studies with a more homogeneous population are warranted.

## CONCLUSION

This study suggests that elevated glutamine in the CSF may be associated with MMD pathogenesis, which differed from ACVD. Taurine in B-MMD and U-MMD was higher than that of ACVD, but the significance was limited due to the difference in dietary supplementation of taurine and age at diagnosis. No difference at the metabolite level was noted between B-MMD and U-MMD. Investigation about MMD-specific metabolites in a larger number of patients is necessary.

## ACKNOWLEDGMENTS

We would like to thank Sung-Eun Kim for her help with the data collection and valuable contribution to the statistical analysis.
